# Comparative genomics highlight the importance of lineage-specific gene families in evolutionary divergence of the coral genus, *Montipora*

**DOI:** 10.1186/s12862-022-02023-8

**Published:** 2022-05-27

**Authors:** Yuki Yoshioka, Go Suzuki, Yuna Zayasu, Hiroshi Yamashita, Chuya Shinzato

**Affiliations:** 1 Atmosphere and Ocean Research Institute, The University of Tokyo, Kashiwa, Chiba Japan; 2 Graduate School of Frontier Science, The University of Tokyo, Kashiwa, Chiba Japan; 3 Fisheries Technology Institute, Japan Fisheries Research and Education Agency, Ishigaki, Okinawa Japan; 4 Marine Genomics Unit, Okinawa Institute of Science and Technology Graduate University, Onna, Okinawa Japan

**Keywords:** Corals, *Montipora*, Comparative genomics, Evolution, Rapidly evolving genes, Symbiosis, Transcriptome, Vertical transmission of algal symbionts

## Abstract

**Background:**

Scleractinian corals of the genus *Montipora* (Anthozoa, Cnidaria) possess some unusual biological traits, such as vertical transmission of algal symbionts; however, the genetic bases for those traits remain unknown. We performed extensive comparative genomic analyses among members of the family Acroporidae (*Montipora*, *Acropora,* and *Astreopora*) to explore genomic novelties that might explain unique biological traits of *Montipora* using improved genome assemblies and gene predictions for *M. cactus*, *M. efflorescens* and *Astreopora myriophthalma*.

**Results:**

We obtained genomic data for the three species of comparable high quality to other published coral genomes. Comparative genomic analyses revealed that the gene families restricted to *Montipora* are significantly more numerous than those of *Acropora* and *Astreopora*, but their functions are largely unknown. The number of gene families specifically expanded in *Montipora* was much lower than the number specifically expanded in *Acropora*. In addition, we found that evolutionary rates of the *Montipora-*specific gene families were significantly higher than other gene families shared with *Acropora* and/or *Astreopora*. Of 40 gene families under positive selection (Ka/Ks ratio > 1) in *Montipora*, 30 were specifically detected in *Montipora-*specific gene families. Comparative transcriptomic analysis of early life stages of *Montipora*, which possesses maternally inherited symbionts, and *Acropora*, which lacks them, revealed that most gene families continuously expressed in *Montipora*, but not expressed in *Acropora* do not have orthologs in *Acropora.* Among the 30 *Montipora*-specific gene families under positive selection, 27 are expressed in early life stages.

**Conclusions:**

Lineage-specific gene families were important to establish the genus *Montipora*, particularly genes expressed throughout early life stages, which under positive selection, gave rise to biological traits unique to *Montipora.* Our findings highlight evolutionarily acquired genomic bases that may support symbiosis in these stony corals and provide novel insights into mechanisms of coral-algal symbiosis, the physiological foundation of coral reefs.

**Supplementary Information:**

The online version contains supplementary material available at 10.1186/s12862-022-02023-8.

## Background

Coral reefs are the most biologically diverse shallow water marine ecosystems [1]. Reef-building corals and endosymbiotic algae of the family Symbiodiniaceae, photosynthetic products of which provide host corals with energy and nutrients, establish mutualistic relationships that are fundamental to coral reefs [2–4]. However, reef-building corals have declined in recent decades due to a variety of anthropogenic stresses, including ocean warming associated with climate change [5–7]. These stresses result in coral bleaching (the breakdown of the symbiosis between corals and their algal endosymbionts [8]), which ultimately leads to loss of habitat for numerous marine species and can precipitate the collapse of entire coral reef ecosystems [9].

**Fig. 1:**
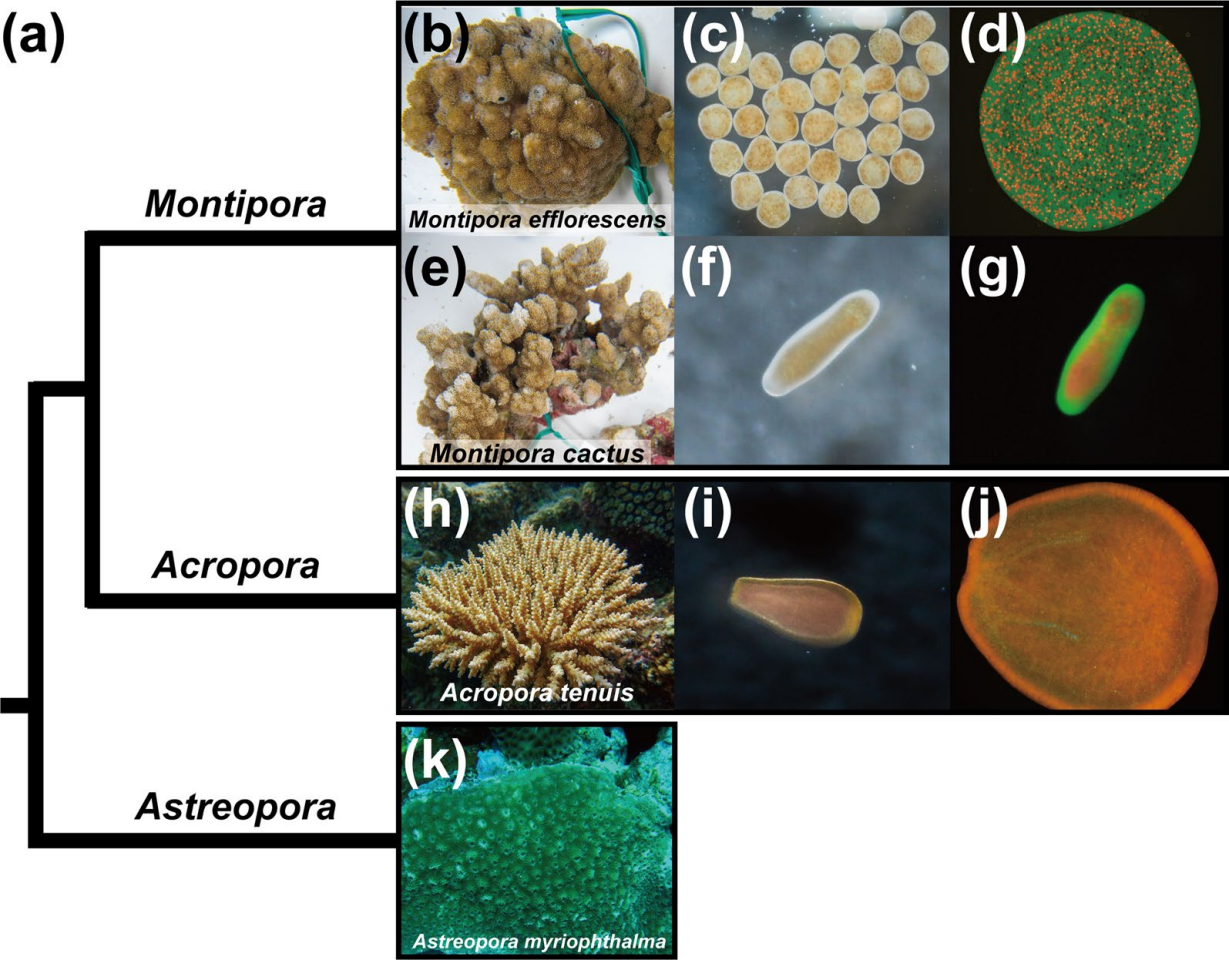
Phylogenetic relationships in the Acroporidae and acroporid morphology. **a** Schematic phylogenetic tree representing evolutionary relationships within the Acroporidae. **b** and **e** Colonies of *Montipora efflorescens* (**b**) and *M. cactus* (**e**). **c** and **d** Eggs of *M. efflorescens* with algal symbionts photographed under visible light (**c**) and blue light (**d**). **f** and **g** A planula larva of *M. efflorescens* with algal symbionts, photographed under visible light (**f**) and blue light (**g**). **h** A colony of *Acropora tenuis*. **i** and **j** A planula larva of *A. tenuis* without algal symbionts photographed under visible light (**i**) and blue light (**j**). **k** A colony of *Astreopora myriophthalma*. Algal symbionts (brown dots) in eggs and planula larvae of *Montipora* (**c** and **f**). Green fluorescence was from fluorescent proteins of *Montipora* and red fluorescence was from chlorophyll in algal symbionts (**d** and **g**). Orange and cyan-green fluorescence were from fluorescent proteins of *Acropora* (**j**)

The genus *Montipora* (family Acroporidae; Fig. 1) is one of the most widespread genera of reef-building corals in the Indo-Pacific [10]. Colony morphology in the genus varies from submassive to laminar, encrusting, and branching colonies [10, 11]. *Montipora* has some unusual and interesting biological traits among acroporid corals, such as maternal transmission of symbionts and higher stress tolerance. Symbiont transmission maintains symbioses across generations and strongly influences host evolution and adaptation to environments [12–14]. Two fundamental symbiont transmission modes predominate in nature (reviewed in [14]): horizontal transmission (symbionts acquired from the environment) and vertical transmission (symbionts acquired maternally). While most coral species (~ 71%), including *Acropora*, acquire symbionts from the ocean in each generation [15], all known *Montipora* species acquire algal symbionts vertically [16, 17] (Fig. 1). Offspring of horizontal recipients generally associate with a broad range of symbiont types and acquire optimal symbionts from new environments [18, 19]; however, there is no guarantee that optimal symbionts will be available. By contrast, offspring of vertical recipients inherit symbionts that are suitable for their physiology [20], but if they encounter an environment that differs significantly from that of their parents, or if the environment changes too drastically, the inherited symbionts may be disadvantageous. *Montipora* also exhibits low sensitivity to ocean acidification and thermal stressors compared to other coral species [21, 22]. These distinct differences between *Montipora* and its close relative, *Acropora*, may have occurred after their divergence (approx. 125 Mya [23]).

In the family Acroporidae, whole-genome data are becoming more readily available, now including 16 species of *Acropora* [23–26], 3 species of *Montipora* [23, 27, 28] and 1 *Astreopora* species [23], the latter being the sister taxon of the remainder of the Acroporidae [29] (Fig. 1). Recently, Shinzato et al. [23] performed a large-scale genomic comparison of acroporids (using genomes of *Acropora*, *Montipora,* and *Astreopora*) and proposed that the evolutionary success of *Acropora* may have resulted from gene duplications. Although some studies have performed genome-wide analysis using *Montipora* genomes [27, 28], the genomic basis for their unique biological traits remains unknown. Exploiting abundant acroporid genomic resources, we performed comparative genomic analyses using improved genomic data of *Montipora* and *Astreopora*. We further identified genes with high evolutionary rates in *Montipora* that may be associated with adaptive evolution, and we specifically attempted to identify genes related to maintenance of maternally inherited symbionts by comparing gene expression during early life stages of *Montipora* and *Acropora*.

## Results

### Improvement of genome assemblies and gene predictions for *Montipora* and *Astreopora*

Assembly error, including retention of allelic contigs in haploid assemblies, is problematic for downstream analyses, mainly due to redundant genome sequences (alleles from the same genetic locus). We curated scaffold sequences of *M. cactus* and *M. efflorescens* by removing scaffold sequences with high or low coverage and those that may have originated from one of two allelic copies in heterozygous regions. Numbers of scaffold sequences were significantly reduced from the previous version, from 4925 to 3521 in *M. cactus* and from 5162 to 3589 in *M. efflorescens* (Table 1). For *Astreopora*, possible allelic scaffold sequences were removed from the genome assembly during the previous study [23]. The previous version of gene models for *M. cactus*, *M. efflorescens*, and *Astreopora* were predicted using AUGUSTUS, based solely on a training set built for *Acropora* or for protein homology with gene models of other corals [23]. Thus, it was highly possible that lineage-specific genes were missed in the previous version. In this study, we performed gene prediction for *M. cactus*, *M. efflorescens*, and *Astreopora myriophthalma* using a combination of ab initio and RNA-seq evidence-based prediction. We predicted 29,158 protein-coding genes for *M. cactus*, 29,424 for *M. efflorescens* and 25,406 for *Astreopora myriophthalma* (Table 1). Benchmarking universal single-copy orthologs (BUSCO) completeness scores were 93.3% (of which 0.8% were duplicated) for *M. cactus*, 91.2% (of which 1.4% were duplicated) for *M. efflorescens* and 94.5% (of which 1.3% were duplicated) for *Astreopora myriophthalma*, which were considerably better scores than the previous version (Table 1). In comparison to other *Montipora* gene models, gene models reported by Shumaker et al. [28] may have contained a higher fraction of diploid copies (93.4% complete BUSCO, with 18.3% duplicated; Table1). Completeness of gene models reported by Helmkampf et al. [27] was lower than that reported by Shumaker et al. [28] (64.2%, with 0.5% duplicated; Table1). Thus, the gene models reported by Shumaker et al. [28] contained many duplicates, but those reported by Helmkampf et al. [27] lacked many genes. In contrast, BUSCO completeness scores of *M. cactus*, *M. efflorescens* and *Astreopora myriophthalma* reported in this study were comparable to published gene models of other coral species, including *A. millepora*, predicted using the NCBI annotation pipeline (Table 1). These improvements to the *Montipora* and *Astreopora* genomes enabled more accurate comparative genomics among acroporids.

**Table 1:** Statistics for gene prediction and assembly of *Montipora, Astreopora* and other coral genomes

Genus	*Montipora*						*Astreopora*		*Acropora*		
Species	*M. cactus*	*M. cactus*	*M. efflorescens*	*M. efflorescens*	*M. capitata*	*M. capitata*	*A. myriophthalma*	*A. myriophthalma*	*A. tenuis*	*A. digitifera*	*A. millepora*^a^
References	This study	Shinzato et al. [23]	This study	Shinzato et al. [23]	Shumaker et al. [28]	Helmkampf et al. [27]	This study	Shinzato et al. [23]	Shinzato et al. [23]	Shinzato et al. [23]	Ying et al. [25]
***Genome assembly***											
Assembly length	640,828,888	65,27,28,006	63,05,35,801	64,33,12,941	88,57,04,498	61,45,09,607	37,34,03,447	37,34,03,447	40,30,90,051	41,58,26,927	38,65,99,652
No. of scaffolds	3532	4925	3589	5162	3043	27,865	1149	1149	1538	955	3869
N50	9,38,091	8,98,511	11,68,433	11,32,316	5,40,623	1,85,537	16,34,277	16,34,277	11,65,818	18,56,312	4,94,532
***Genes***											
No. of protein coding genes	29,158	21,983	29,424	21,370	63,227	36,691	25,406	27,576	22,905	22,327	23,710
Avg. length	12,905	15,343	12,546	15,531	6507^b^	4048^b^	9550	5503^b^	10,298	10,445	9294
***Transcripts***											
Avg. no. of CDS (exon) per gene	6.6 (7.2)	7.7 (7.9)	6.5 (7.1)	7.8 (8.0)	4.5 (NA)	3.62 (NA)	6.0 (6.4)	5.0 (NA)	7.2 (8.1)	6.6 (8,5)	7.2 (7.5)
Avg. CDS (exon) length per gene (bp)	1411 (2,033)	1871 (2139)	1390 (1994)	1876 (2143)	1162 (NA)	1103 (NA)	1647 (2132)	1426 (NA)	1562 (2383)	1435 (2415)	1600 (2404)
Avg. CDS (exon) length	215 (280)	244 (271)	215 (280)	241 (270)	259 (NA)	305 (NA)	230 (279)	283 (NA)	216 (294)	217 (285)	312 (317)
Tortal CDS (exon) length (Mbp)	41 (59)	41 (45)	41 (59)	40 (44)	73 (NA)	40 (NA)	42 (54)	41 (NA)	36 (55)	32 (54)	38 (57)
***BUSCO completeness (v5.0)***											
Complete	93.3%	87.9%	91.2%	84.1%	93.4%^b^	64.2%^b^	94.5%	78.4%^b^	92.9%	89.3%	94.7%
Single-copy	92.5%	87.3%	89.8%	83.6%	75.1%^b^	63.7%^b^	93.2%	77.7%^b^	92.2%	88.6%	93.7%
Duplicated	0.8%	0.6%	1.4%	0.5%	18.3%^b^	0.5%^b^	1.3%	0.7%^b^	0.7%	0.7%	1.0%
Fragmented	4.1%	5.7%	4.5%	7.2%	4.1%^b^	13.5%^b^	3.1%	7.7%^b^	2.9%	4.7%	2.7%
Missing	2.6%	6.4%	4.3%	8.7%	2.5%^b^	2.5%^b^	2.4%	13.9%^b^	4.2%	6.0%	2.6%

^a^RefSeq assembly (GCF_004143615.1) was used for the analysis

^b^CDS were used for the calculation

### Comparison of gene families within the Acroporidae

**Fig. 2:**
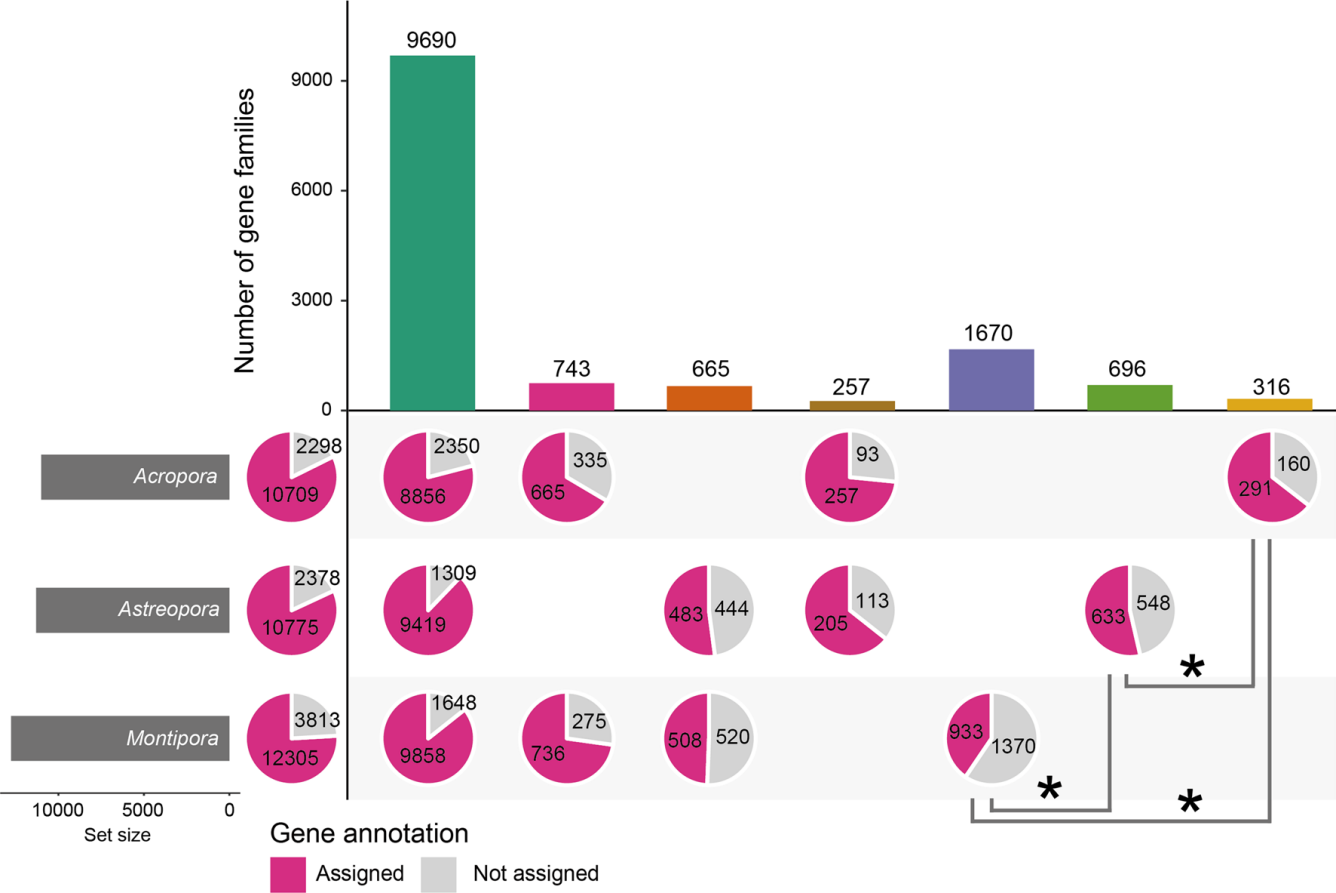
Gene family composition in acroporid genomes and the higher proportion of genes in *Montipora* having unknown functions. Left horizontal bars indicate numbers of gene families in each genus. Vertical bars indicate numbers of gene families conserved among genera. Pie charts indicate the generic composition in a given number of gene families (vertical bars). Gene annotation was performed using BLAST searches against the Swiss-Prot database (BLASTP, E-value cutoff: 1e^−5^) and protein domain search with hidden Markov models against the Pfam database (InterProScan, E-value cutoff: 1e^−3^). Numbers in pie charts indicate the number of genes with similarity to either Swiss-Prot or Pfam or neither. Proportions of gene annotations were compared among gene families specific to each lineage and asterisks indicate statistical significance (Pairwise proportion test: *p* < 0.05). Upset plot was produced using the “UpSetR” package [79]

Identifying orthologous relationships between sequences is fundamental for comparative genomic analyses. To obtain orthologous relationships among acroporid genomes, we used three *Acropora* species (*A. digitifera*, *A. millepora*, and *A. tenuis*), two *Montipora* species (*M. cactus* and *M. efflorescens*), and *Astreopora myriophthalma*. We obtained 12,769 gene families for *Montipora*, 11,007 for *Acropora* and 11,309 for *Astreopora* (Fig. 2). We then categorized each gene family into seven groups, (1) shared by all three genera (9690 gene families), (2) shared by *Montipora* and *Acropora* (743 gene families), (3) shared by *Montipora* and *Astreopora* (665 gene families), (4) shared by *Acropora* and *Astreopora* (257 gene families)*,* (5) restricted to *Montipora* (1670 gene families), (6) restricted to *Astreopora* (696 gene families) and (7) restricted to *Acropora* (316 gene families) (Fig. 2). 75.8% (9690/12,769) of the gene families in *Montipora*, 88% (9690/11,007) in *Acropora*, and 85.7% (9690/11,309) in *Astreopora* were shared among all three genera (Fig. 2), indicating that a large number (~ 88%) of gene families are shared throughout the Acroporidae, and these are likely to be the core-gene families among acroporids (Additional file 9: Data S1).

The two major clades of reef-building corals, known as Robusta and Complexa [30], possess different metabolic pathways [31]. From the six species, we compared 303 functional modules comprising ten categories in the Kyoto Encyclopedia of Genes and Genomes (KEGG) metabolic pathways and found that metabolic pathways were basically conserved in the three genera (Additional file 1: Table S1). An enzyme involved in cysteine biosynthesis (KEGG module ID: M00338) and methionine degradation (KEGG module ID: M00035) was not detected among the six species (Additional file 1: Table S1), as reported in Shinzato et al. [23, 24]. Although one gene (KEGG entry ID: K04486) involved in the histidine biosynthetic pathway (KEGG module ID: M00026) was detected in acroporid corals used in this study, the remaining genes required to complete the pathway were not detected (Additional file 1: Table S1), as reported in Ying et al. [31]. Taken together, gene families involved in common features, such as amino acid synthesis, are widely conserved in the three genera.

While we identified 696 lineage-specific gene families in *Astreopora* and 316 in *Acropora*, we identified 1670 gene families restricted to *Montipora* (2307 genes in *M. cactus* and 2303 in *M. efflorescens*) (Fig. 2). The proportion of lineage-specific gene families in *Montipora* (13.07%) was significantly larger than that in *Acropora* (2.87%) and *Astreopora* (6.15%) (Pairwise proportion test: *p* < 0.05). Although we performed gene annotation with BLAST searches against the Swiss-Prot database (BLASTP, E-value cutoff: 1e^−5^) and hidden Markov models against the Pfam database (InterProScan, E-value cutoff: 1e^−3^), the proportion of *Montipora*-specific gene families with gene annotation was significantly lower than in *Acropora* and *Astreopora* (Pairwise proportion test: *p* < 0.05 for all combinations; Fig. 2). This indicates that functions of gene families restricted to *Montipora* are largely unknown.

### Gene expansions in* Montipora *and comparisons among acroporids

**Fig. 3:**
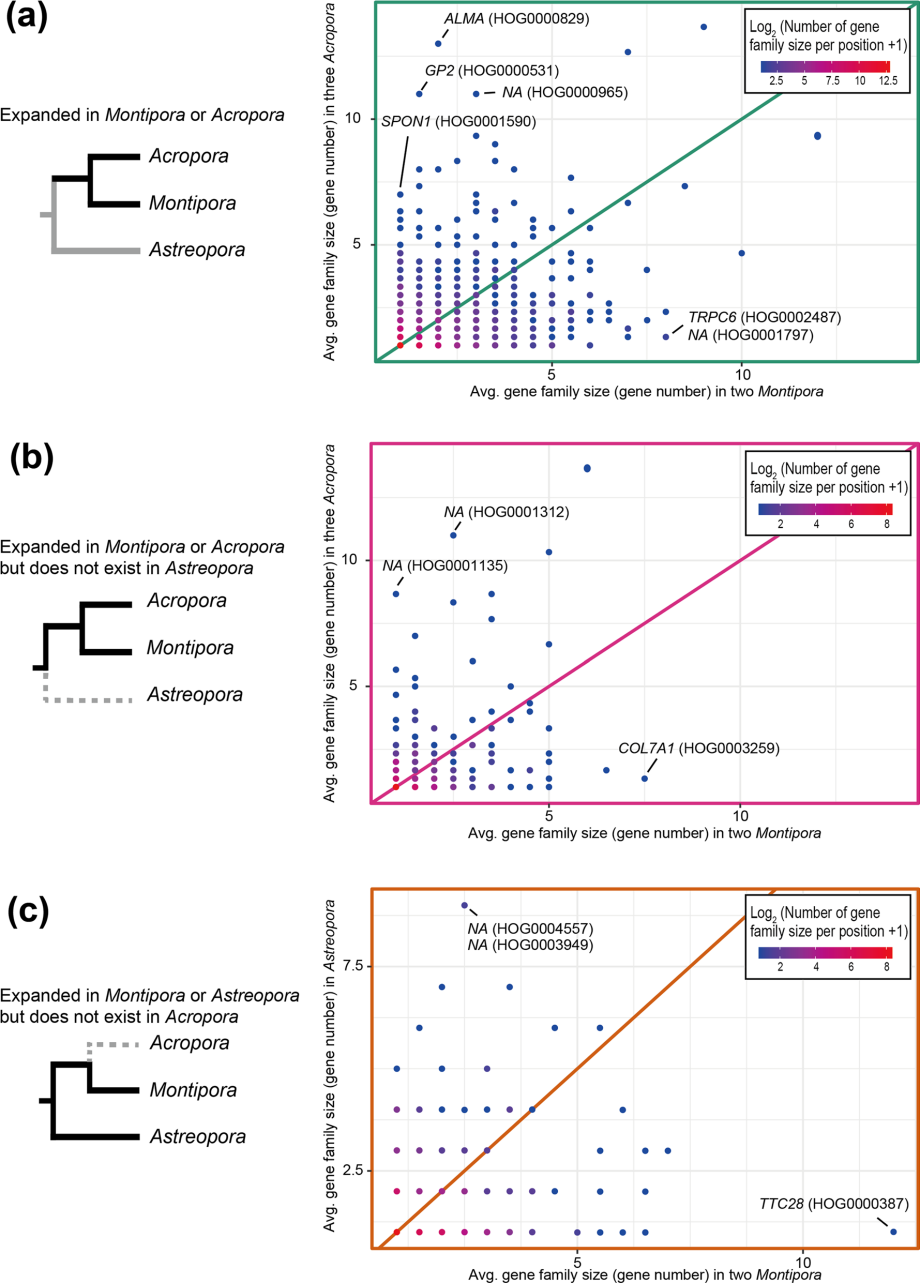
Gene family expansions in *Montipora*. **a** Numbers of genes in *Montipora* and *Acropora* in each gene family shared by the three genera. **b** Numbers of genes in *Montipora* and *Acropora* in each gene family shared by *Montipora* and *Acropora*. **c** Numbers of genes in *Montipora* and *Astreopora* in each gene family shared by *Montipora* and *Astreopora*. The diagonal solid line indicates 1:1 numbers of genes in orthologous families. Possible gene names and gene family IDs are shown for significantly expanded gene families (Fisher’s exact test: *p* < 0.05). Scatter plot was produced using the “ggplot2” package [80]

Gene duplication has contributed to acquisition of new gene functions during evolution [32, 33]. To explore gene families that underwent expansions, we first compared gene numbers of 9,690 gene families shared by the three genera and 743 gene families shared by *Montipora* and *Acropora* (Fig. 2). In these two groups, genes in families that underwent gene expansions in either *Montipora* or *Acropora* might have been duplicated after *Montipora* and *Acropora* diverged from their last common ancestor. Three gene families, homologous dimethylsulfoniopropionate (DMSP) lyase (*ALMA*; HOG0000829), Endonuclease-reverse transcriptase (*GP1*; HOG0000531), and Spondin (*SPON1*; HOG0001590), and three non-annotated gene families (NA; HOG0000965, HOG0001135, and HOG0001312), were significantly expanded in *Acropora* (Fisher’s exact test: *p* < 0.05; Fig. 3a and b). Recently, it was reported that DMSP lyase is the most expanded gene family in *Acropora* [28], and our result is consistent with that report, supporting the accuracy of this analysis. We found that three gene families, transient receptor potential protein (*TRPC*; HOG0002487), collagen alpha-1 (VII) chain (*COL7A1*; HOG0003259) and non-annotated gene family (NA; HOG0001797) are significantly expanded in *Montipora* compared with *Acropora* (Fisher’s exact test: *p* < 0.05; Fig. 3a and 3b).

Next, we compared gene numbers of 665 gene families shared by *Montipora* and *Astreopora* (Fig. 2), in which gene duplication may have occurred after divergence of *Montipora* or *Astreopora*. These genes may have been lost in *Acropora*. Two gene families (HOG0003949 and HOG0004557) lacking Swiss-Prot annotation were significantly expanded in *Astreopora* (Fisher’s exact test: *p* < 0.05; Fig. 3c), whereas one other gene family, tetratricopeptide repeat protein 28 (*TTC28*; HOG0000387), which is involved in the cell cycle in humans [34], was significantly expanded in *Montipora* compared with *Astreopora* (Fisher’s exact test: *p* < 0.05; Fig. 3c).

### Estimation of evolutionary rate in each *Montipora* gene family group

**Fig. 4:**
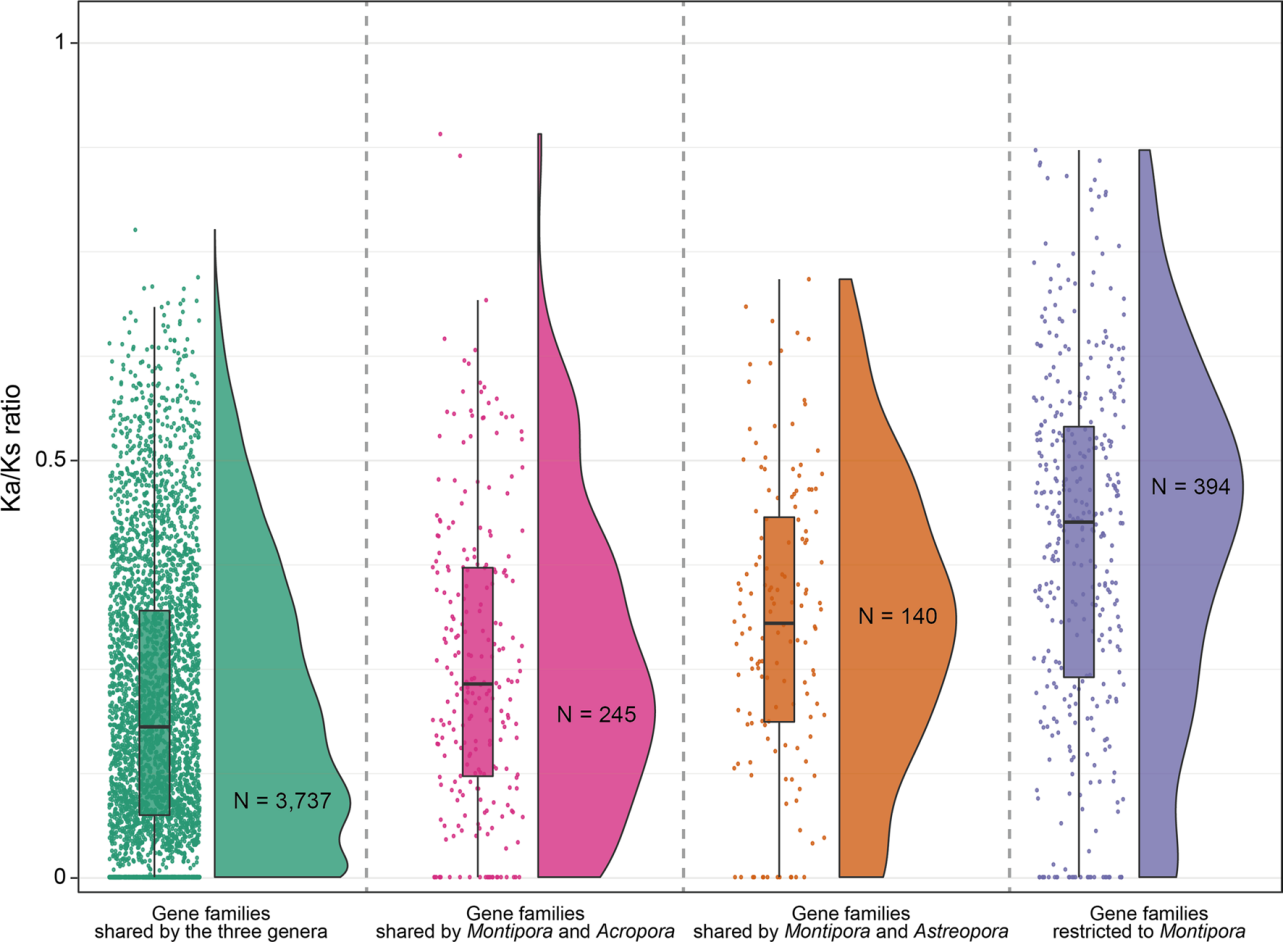
Relaxed purifying selection in *Montipora*-specific gene families. The y-axis represents the ratio of nonsynonymous (Ka) to synonymous amino acid substitutions (Ks). Orthologous gene pairs in two *Montipora* species (*M. cactus* and *M. efflorescens*) are used for calculation of pairwise Ka/Ks ratios. Ka/Ks ratios were compared among gene families and significant differences were observed in all pairwise combinations (Wilcoxon rank sum test: *p* < 0.05). A raincloud plot was produced using the “raincloudplots” package [81]. Details of 40 gene families exhibiting Ka/Ks > 1 are shown in Table 2

The ratio of nonsynonymous (Ka) to synonymous substitutions (Ks) reflects the strength of selective pressure on protein sequences [35]. For example, when Ka is less than Ks (Ka/Ks < 1), selection has occurred to eliminate mutations of protein sequences (purifying selection). In contrast, when Ka is larger than Ks (Ka/Ks > 1), selection has occurred to mutate the protein sequences (positive selection). In order to evaluate the strength of selective pressure acting on protein sequences in each *Montipora* gene family, we calculated pairwise Ka/Ks ratios between *Montipora* single-copy orthologous gene pairs (*M. cactus* versus *M. efflorescens*) for each of the four groups: (1) gene families shared by the three Acroporidae genera, (2) gene families shared by *Montipora* and *Acropora*, (3) gene families shared by *Montipora* and *Astreopora*, and (4) gene families restricted to *Montipora* (Fig. 4). When we compared Ka/Ks ratio between groups, gene families restricted to *Montipora* showed the highest Ka/Ks ratio (Wilcoxon rank sum test: *p* < 0.05; Fig. 4), indicating that this gene family group has undergone a relaxation of purifying selection, and that functional constraints on this gene family group are relaxed. This could explain why the deduced gene functions of gene families restricted to *Montipora* are largely unknown.

### Positive selection specific to *Montipora*

To identify genes with fast evolutionary rates that may be associated with adaptive evolution in *Montipora*, we focused on gene families exhibiting Ka/Ks > 1. We found evidence of positive selection in 40 gene families (rapidly evolving gene families) (Table 2). Of those, 10 families are shared by the three genera or shared by *Montipora* and *Acropora*, while the remaining 30 families are restricted to *Montipora* (Table 2), suggesting that these 30 gene families arose specifically in that lineage and likely contribute to biological traits unique to *Montipora*. Although 28 of the 30 gene families restricted to *Montipora* were without annotation, their possible subcellular localization ranging from membrane to organelle was predicted by DeepLoc, a deep learning neural networks model (Table 2).

**Table 2:** Gene families under positive selection (Ka/Ks ratio > 1) in *Montipora*

Gene family group	Gene family ID	Expressed in early life stages of	Swiss-Prot annotation (BLASTP, e-value cutoff: 1e^−5^)	Pfam domain (InterProScan, e-value cutoff: 1e^−3^)	Subcellular localization	Ka	Ks	Ka/Ks ratio
Common to the three genera	HOG0011297	*Montipora* and *Acropora*	G patch domain and ankyrin repeat-containing protein 1	Ankyrin repeats (3 copies) (PF12796) G-patch domain (PF01585)	Nucleus	1.02946	0.868207	1.18573
	HOG0013033	*Montipora* and *Acropora*	NA	NA	Nucleus	0.047466	0.016942	2.80166
	HOG0013161	*Montipora* and *Acropora*	NA	NA	Extracellular	0.067747	0.011507	5.88723
	HOG0013504	*Montipora* and *Acropora*	NA	NA	Extracellular	0.250122	0.146258	1.71014
	HOG0016452	*Montipora* and *Acropora*	Nucleoredoxin-like protein 2	Thioredoxin-like (PF13905)	Cytoplasm	1.04061	0.866303	1.2012
Common to *Montipora* and *Acropora*	HOG0000399	-	NA	NA	Mitochondrion	1.04494	0.829832	1.25922
	HOG0007354	*Montipora* and *Acropora*	NA	NA	Cytoplasm	0.043171	0.01538	2.80698
	HOG0008211	*Montipora* and *Acropora*	E3 ubiquitin-protein ligase TRIM71	RING-type zinc-finger (PF13445) B-box zinc finger (PF00643)	Nucleus	1.02988	0.901833	1.14199
	HOG0008701	*Montipora* and *Acropora*	Collagen alpha-1(V) chain	Fibrillar collagen C-terminal domain (PF01410)	Extracellular	0.086894	0.031594	2.75035
	HOG0018764	*Montipora and Acropora*	Histamine H2 receptor	7 transmembrane receptor (rhodopsin family) (PF00001)	Cell membrane	1.03386	0.896216	1.15358
Restricted to *Montipora*	HOG0000410	*Montipora*^a^	NA	NA	Cytoplasm	1.0489	0.858182	1.22224
	HOG0006069	*Montipora*^a^	NA	NA	Cytoplasm	1.06207	0.789879	1.34459
	HOG0025744	*Montipora*^a^	NA	NA	Cytoplasm	1.04814	0.846526	1.23816
	HOG0025248	*Montipora*^a^	NA	NA	Golgi apparatus	1.06112	0.837214	1.26744
	HOG0025123	*Montipora*^a^	NA	NA	Mitochondrion	1.03568	0.871894	1.18785
	HOG0025167	*Montipora*^a^	NA	NA	Mitochondrion	1.05341	0.81252	1.29647
	HOG0025503	*Montipora*^a^	NA	NA	Mitochondrion	1.06094	0.794999	1.33451
	HOG0025176	*Montipora*^a^	NA	NA	Plastid	1.07662	0.736763	1.46128
	HOG0025663	*Montipora*^a^	NA	NA	Lysosome/Vacuole	1.0435	0.883964	1.18048
	HOG0023383	*Montipora*^b^	NA	NA	Cytoplasm	1.0506	0.86614	1.21297
	HOG0024846	*Montipora*^b^	NA	NA	Cytoplasm	0.157342	0.054572	2.8832
	HOG0025044	*Montipora*^b^	NA	NA	Cytoplasm	1.04722	0.8635	1.21276
	HOG0025199	*Montipora*^b^	NA	NA	Cytoplasm	1.04978	0.833375	1.25967
	HOG0024900	*Montipora*^b^	NA	NA	Endoplasmic reticulum	0.072466	0.030348	2.38782
	HOG0025608	*Montipora*^b^	NA	NA	Endoplasmic reticulum	1.05623	0.836453	1.26275
	HOG0024879	*Montipora*^b^	NA	NA	Extracellular	1.04481	0.820572	1.27328
	HOG0024911	*Montipora*^b^	NA	NA	Extracellular	1.08591	0.764205	1.42097
	HOG0024765	*Montipora*^b^	NA	NA	Mitochondrion	1.06916	0.745005	1.43511
	HOG0025496	*Montipora*^b^	NA	NA	Mitochondrion	1.03657	0.886546	1.16922
	HOG0025423	*Montipora*^b^	NA	NA	Mitochondrion	1.05678	0.799324	1.32209
	HOG0025535	*Montipora*^b^	Integrase/recombinase xerD homolog	NA	Mitochondrion	1.04223	0.880029	1.18431
	HOG0025606	*Montipora*^b^	NA	NA	Mitochondrion	1.05418	0.833955	1.26408
	HOG0024806	*Montipora*^b^	NA	Zinc knuckle (PF00098)	Nucleus	1.0438	0.876216	1.19126
	HOG0024834	*Montipora*^b^	NA	NA	Nucleus	1.04457	0.853951	1.22323
	HOG0024972	*Montipora*^b^	NA	NA	Nucleus	1.06879	0.737384	1.44943
	HOG0025048	*Montipora*^b^	NA	NA	Nucleus	1.0465	0.828206	1.26357
	HOG0025616	*Montipora*^b^	NA	NA	Nucleus	1.07293	0.838674	1.27932
	HOG0024996	-	NA	NA	Cell membrane	0.827813	0.407974	2.02908
	HOG0024975	-	NA	NA	Endoplasmic reticulum	1.02887	0.884795	1.16284
	HOG0025445	-	TNF receptor-associated factor 6	TRAF-type zinc finger (PF02176)	Nucleus	0.094242	0.019425	4.85163

^a^Expressed at least one early life stages

^b^Expressed at all three early life stages

### Gene expression unique to early life stages of *Montipora*

**Fig. 5:**
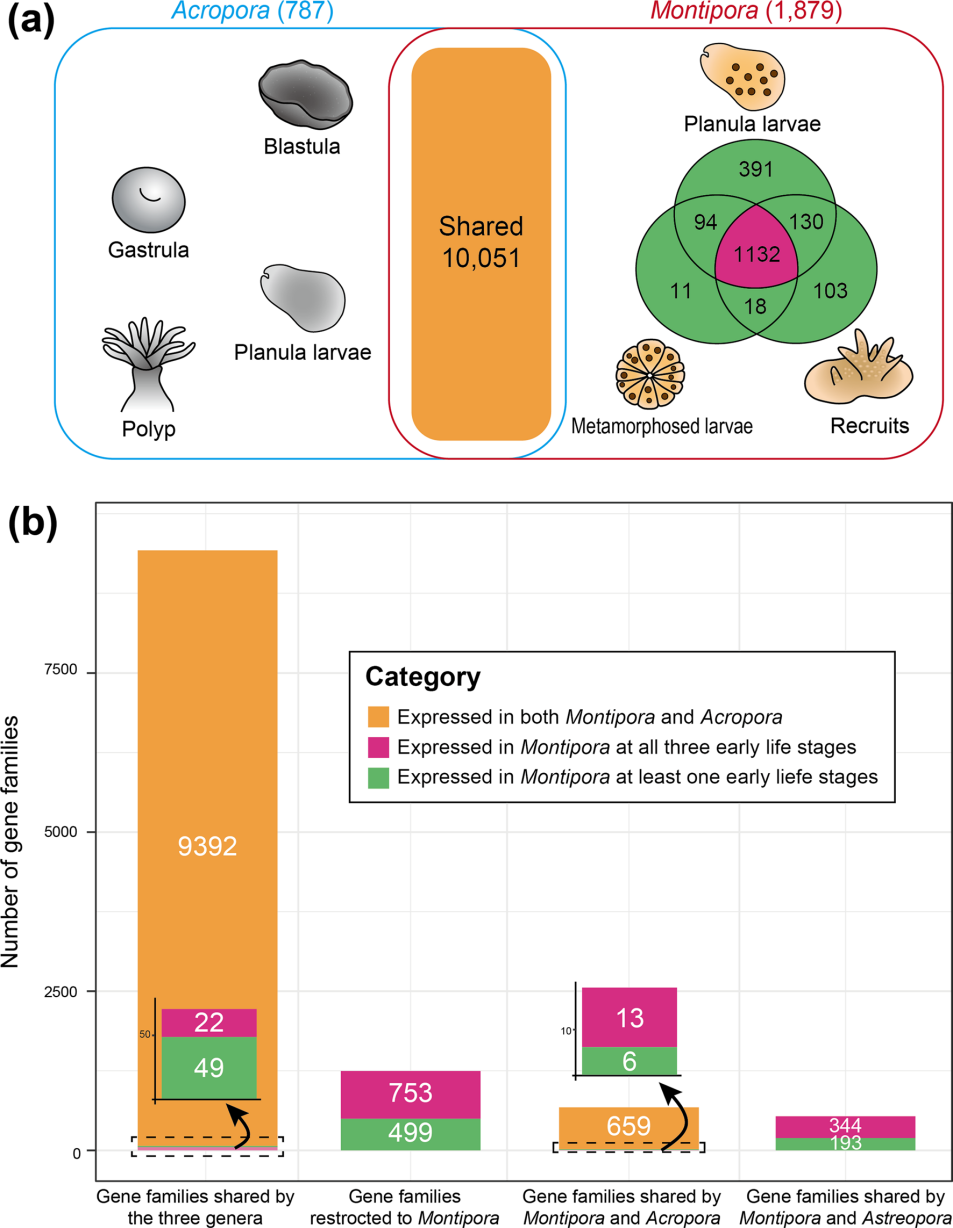
Expression patterns of gene families during early life stages of *Montipora and Acropora*. **a** Numbers of gene families that are commonly or exclusively expressed in early life stages of *Montipora* and *Acropora*. Numbers of gene families that are exclusively expressed in each genus are shown in parentheses after generic names. Gene families that are commonly expressed in both *Montipora* and *Acropora* early life stages are shown in an orange box. Gene families that are exclusively expressed in early life stages of *Montipora* were further classified according to whether they are expressed in one of the stages (green), or throughout all stages (red). **b** Gene families expressed in early *Montipora* life stages. For *Montipora*, RNA-seq data from planula larvae, metamorphosed larvae and recruits were used. For *Acropora*, RNA-seq data from blastulae, gastrulae, planula larvae and polyps were used. SRA accession numbers for the RNA-seq data are provided in Additional file 7: Table S7

Presence of maternally inherited algal symbionts at an early life stage is the most obvious difference between vertical and horizontal transmitters (Fig. 1). In order to identify gene families specifically involved in symbiosis in vertical transmitters, we compared the repertoire of expressed genes in early life stages of *Montipora* with those expressed in *Acropora*. In this analysis, a gene family was considered expressed even if only one gene in that family was expressed (Transcripts per million (TPM) > 1). We confirmed that 11,930 and 10,838 gene families were expressed during early life stages of *Montipora* and *Acropora*, respectively (Fig. 5a). Of these, 10,051 gene families (84% in *Montipora* and 93% in *Acropora*) were shared by both during early life stages (Fig. 5a), suggesting that these are essential for early development of acroporid corals; thus, we did not focus on these in the present study. We identified 1,879 gene families that were exclusively expressed in *Montipora* (Fig. 5b). Among those, 60% (1132 gene families) were expressed in planula larvae, metamorphosed larvae, and recruit stages (Fig. 5a), suggesting that these genes may be related to maintenance of algal symbionts in *Montipora*. Interestingly, 97% of these gene families ((753 + 344) / 1132, Fig. 5b) that were expressed throughout the three life stages were specific to *Montipora* or shared by *Astreopora* (Additional file 2: Table S2). In contrast, the remaining 3% of gene families ((22 + 13) / 1,132, Fig. 5b) have orthologs in *Acropora*, but were not expressed in *Acropora*. Nonetheless, they were expressed throughout early life stages of *Montipora* Additional file 3: Table S3). Within gene families containing gene duplications in the *Montipora* genomes above, two gene families (HOG0001797 and HOG0000387) were exclusively expressed in at least one early life stage in *Montipora*, and one of them (HOG0000387) was expressed throughout all three early life stages (Additional file 2: Table S2). Among the identified 30 rapidly evolving gene families restricted to *Montipora*, we detected gene expression of 90% of these families. Expression of nine families was detected in at least one early life stage of *Montipora*, and the remaining 18 gene families were continuously expressed throughout all three early life stages (Table 2).

## Discussion

### Improved genome information for the genera *Montipora* and *Astreopora*

BUSCO completeness scores for improved gene models of *M. cactus*, *M. efflorescens*, and *Astreopora myriophthalma* were 93.3% (0.8% duplicates), 91.2% (1.4% duplicates), and 94.5% (1.3% duplicates), respectively (Table 1). They are considerably better than those of *M. capitata* (93.4% (18.3% duplicates) from Shumaker et al. [28] and 64.2% (0.5% duplicates) from Helmkampf et al. [27]; Table 1), and were comparable to those of other coral species (Table 1). These numbers indicate that we successfully obtained high-quality gene models from *Montipora* and *Astreopora* species. Numbers of genes in *M. cactus and M. efflorescens* genomes were not quite as large as those of *M. capitata* reported by Shumaker et al. [28]. Previously, it was reported that *M. capitata* has fewer exons and shorter coding regions per gene than other corals [27, 28]; however, this was not the case with *M. cactus* and *M. efflorescens* (Table 1). Fewer exons and shorter coding regions per gene could be an unusual feature of the *M. capitata* genome or could reflect the quality of the genome assembly. Indeed, the N50 size, one of the indices to evaluate the quality of genome assembly, was larger for both *M. cactus* and *M. efflorescens* genome assemblies than for *M. capitata* (Table 1).

### Possible genomic evolutionary strategy unique to *Montipora*

Recent large-scale genome comparisons of acroporid genomes showed that 28 gene families were specifically expanded in *Acropora*, but none in *Montipora* [23]. Nonetheless, we identified four expanded gene families in *Montipora* (Fig. 3). Although the number of gene families in *Montipora* is not much different from those of *Acropora* and *Astreopora*, the proportion of lineage-specific gene families in *Montipora* was significantly larger (Fig. 2). *Montipora* does not appear to have duplicated existing gene families, as has *Acropora*. Lineage-specific gene families contribute to larger gene numbers in *Montipora* genomes, and emergence of lineage-specific genes may have helped to establish maternal transmission of symbionts in *Montipora* corals. In particular, *Montipora*-specific gene families under positive selection may be major contributors.

Three gene families, homologous to *TRPC6*, *TTC28,* and *COL7A1*, and one gene family without annotation were significantly expanded in *Montipora* compared with *Acropora* or *Astreopora* (Fig. 3). Known functions of transient receptor potential (TRP) proteins encoded by *TRPC* are diverse (reviewed in [36]). For example, TRP proteins respond to hypertonicity in yeasts [37, 38], detect and avoid noxious chemicals in nematodes [39], and discriminate warmth, heat, and cold in humans [36]. In each case, TRP proteins mediate sensory transduction in cells [36]. In corals, expression levels of *TRP*-like genes change when the concentration of CO_2_ in seawater changes [40]. They also change diurnally [41, 42] or when exposed to symbiotic algae [43, 44]. The *TRPC6*-like gene family, specifically expanded in *Montipora*, may also be involved in sensory transduction during environmental transitions. The *TTC28*-like gene family has tetratricopeptide repeats (PF12176 and/or PF13424) and caspase HetF associated with Tprs (CHAT) domains (PF12770) (Additional file 4: Table S4). Canonical *TTC28* is composed of tetratricopeptide repeats and CHAT domains (Q96AY4: TTC28_HUMAN [34]) and genes in the gene family (HOG0000387) are also composed of tetratricopeptide repeats and CHAT domains, indicating that this gene family may have been duplicated from canonical *TTC28*, which is conserved in all acroporids examined in this study (HOG0016559 in Additional file 9: Data S1). *TTC28* is required for the cell cycle in humans [34]. The expanded *TTC28*-like gene family may also be involved in cell cycle in *Montipora*. Collagen is expressed in gastrodermis at a specific developmental stage of cnidarian larvae [45–47] and the expanded collagen-like gene family may function in early development of *Montipora*.

In this study, we identified 40 genes under positive selection in *Montipora* (Table 2). Positive selection has often been detected in genes involved in immunity in vertebrates [48]. In corals, genes related to immunity, such as lectins and antimicrobial peptides, are also under positive selection [23, 49, 50]. In the 40 rapidly evolving gene families found in this study, with one exception, no genes appeared homologous to immune-related genes (Table 2). In addition, 28 of 30 rapidly evolving gene families restricted to *Montipora* have no annotated function (Table 2). Generally, genes with no homology to genes of other lineages are called orphan genes [51]. They are thought to arise principally by two processes: gene duplication or de novo evolution from non-coding regions [51]. If a gene originates by duplication, the protein domains tend to be conserved in the new genes, since a functional protein domain cannot easily be changed by mutations [52], suggesting that the 28 rapidly evolving gene families originated by de novo evolution from non-coding regions. Orphan genes are expected to interact specifically with the environment as a consequence of lineage-specific adaptation [51]. Therefore, orphan genes may contribute to adaptive evolution in *Montipora*. In particular, 18 rapidly evolving gene families with expression throughout the three early life stages, planula larvae, metamorphosed larvae, and recruits, may have important functions in symbiosis during early life stages of *Montipora*.

## Conclusions

In this study, we highlighted possible genomic underpinnings of maternal transmission of symbionts in *Montipora* using high-quality genomic information of *Montipora* and *Astreopora*. We found that the driving force behind evolution of *Montipora* was lineage-specific gene families, rather than gene duplication, as among *Acropora* corals. The importance of rapidly evolving gene families in *Montipora* for maternal transmission of symbionts is inferred. Our dataset and findings offer novel insights into mechanisms of coral-algal symbiosis. Although genetic tools for manipulating corals have been established [53, 54], development of more efficient methods to deliver gene-knockdown or -knockout reagents into large numbers of zygotes will facilitate rapid screening for relevant phenotypes of candidate genes. In addition, coral cell lines which have the capacity to incorporate algal symbionts has been developed [55], allowing us to observe ongoing symbiosis at the single cell level. Together, these advances will facilitate a deeper understanding of cellular and molecular mechanisms of coral-algal symbiosis.

## Methods

### Sample preparation, RNA extraction, and RNA-Seq

Colonies of *M. cactus, M. efflorescens,* and *Astreopora myriophthalma* were collected in Sekisei Lagoon, Okinawa, Japan in May 2015, and were maintained in aquaria at the Research Center for Subtropical Fisheries, Seikai National Fisheries Research Institute, until spawning. Permits for coral collection were kindly provided by the Okinawa Prefectural Government for research use (Permits #29-74). Coral fragments (~ 3 cm diameter) from adult colonies of *M. cactus*, *M. efflorescens*, and *Astreopora myriophthalma* were snap frozen in liquid nitrogen and stored at -80℃ until use. Fragments were then crushed in liquid nitrogen with an iron and hammer into powder. Total RNA was extracted from the powder using an RNeasy Plant Mini Kit (QIAGEN). A TruSeq Stranded mRNA Library Kit (Illumina) was used for mRNA sequencing library preparation, and each library was sequenced from 100-bp paired-end libraries using a NovaSeq 6000 (Illumina). For *Montipora*, eggs, sperm, planula larvae (1 and 4 d post-fertilization) were collected and preserved with TRIzol reagent (Thermo Fisher Scientific) at -80℃ until use. Total RNA was extracted from preserved eggs, sperm, and planula larvae as in Yoshioka et al. [56]. KAPA RNA HyperPrep Kits (Kapa Biosystems) and MGIEasy RNA Directional Library Prep Sets (MGI) were used for total RNA and mRNA sequencing library preparation, and each library was sequenced on a NovaSeq 6000 in 150-bp paired-end and a DNBSEQ-G400RS (MGI) in 100-bp paired-end mode. This information is summarized in Additional file 5: Table S5.

### Curating scaffold sequences of *M. cactus* and *M. efflorescens* and gene prediction

We downloaded scaffold sequences of *M. cactus* and *M. efflorescens,* assembled from DNA sequences extracted from symbiotic algae-free coral sperm [23], from the genome browser of the OIST Marine Genomics Unit (https://marinegenomics.oist.jp). We identified scaffold sequences with high or low coverage or those that may have originated from one of two allelic copies of heterozygous regions, using Purge Haplotigs v1.1.1 [57] with default settings. These were excluded from subsequent analyses.

In addition to the above RNA samples, we used publicly available RNA-seq data from NCBI SRA for gene prediction (Additional file 6: Table S6). Low-quality reads (quality score < 20 and length < 20 bp) and sequence adaptors were trimmed using CUTADAPT v1.18 [58]. A total of 31 and 2 RNA-seq libraries were used for *Montipora* and *Astreopora* gene prediction, respectively. Repetitive elements in the scaffolds were identified de novo with RepeatScout v1.0.6 [59] and RepeatMasker v4.1.0 (http://www.repeatmasker.org). Repetitive elements were filtered out by length (> 50 bp) and occurrence (more than 10 times for *Montipora*, more than 60 times for *Astreopora*). Gene prediction was first executed with the BRAKER pipeline v2.1.2 [60], with AUGUSTUS v3.3.3. RNA-seq reads were aligned to each genome sequence with HISAT v2.1.0 [61]. Then, the alignment information was used for BRAKER gene prediction with options “UTR = on”, “softmasking”, and “AUGUSTUS_ab_initio.” To improve gene prediction, we further executed genome-guided transcriptome assembly using StringTie [62] with option “-m 500.” Genome-based transcript structure was predicted with TransDecoder (https://github.com/TransDecoder/TransDecoder/wiki). During read alignment, we used soft-masked repeats for genome-guided transcriptome assembly and hard-masked repeats for BRAKER gene prediction. Finally, genes that were present in genome-guided assembly or ab initio prediction, but absent in predictions from the hint file were added to the prediction from the hint file using GffCompare [63], as summarized in Additional file 8: Figure S1. To evaluate the completeness of predicted genes, we used BUSCO v5.0 [64] with Metazoa OrthoDB10 dataset (2021–02-24, n = 954).

### Gene annotation, orthology inference within the Acroporidae

We used publicly available gene models for *A. digitifera* [23, 24], *A. tenuis* [23], and *A. millepora* [25] in addition to *Montipora* and *Astreopora* gene models. For *A. millopora*, we downloaded gene models from NCBI RefSeq (RefSeq assembly accession: GCF_004143615.1). We downloaded gene models of v2.0 for *A. digitifera* and v1.0 for *A. tenuis* from the genome browser of the OIST Marine Genomics Unit, respectively. We selected three *Acropora* species for the following reasons. *A. digitifera* genome sequences were assembled with PacBio long-reads [23]. *A. millepora* genome assembly and gene models were curated by NCBI. *A. tenuis* had the second highest gene model completeness (BUSCO completeness scores) among *Acropora* genomes following *A. millepora* [23] and represents a distinct phylogenetic clade from *A. digitifera* and *A. millepora* [23]*.* We selected the longest transcript variants from each gene and translated them into protein sequences. All proteomes were annotated with BLASTP [65] (E-value cut off: 1e^−5^) against the Swiss-Prot database (8 January 2021). In addition, domains in protein sequences were searched using hidden Markov models against the Pfam database with InterProScan v5.31–70.0 (E-value cutoff: 1e^−3^) [66]. In addition, putative transposable elements in gene models were identified with TransposonPSI (http://transposonpsi.sourceforge.net/), Dfam scan (release 3.3; Storer et al. [67]), and Pfam keyword (“Reverse transcriptase” and “Integrase”). All protein sequences were also annotated with KEGG [68] in all eukaryote genes using GenoMaple v2.3.2 [69] with the GHOSTX search engine and the bi-directional best hit method. Module completion ratio (MCR) was calculated in each functional module defined by KEGG, also using GenoMaple v2.3.2. For clustering of orthologous genes (herein gene families) of the Acroporidae, we used OrthoFinder v2.4.0 [70] and *Porites australiensis* gene models [71] were also included as an outgroup for the Acroporidae. In this study, we used phylogenetic hierarchical orthogroups (HOG) as gene families. Gene families shared by the three *Acropora* species were defined as *Acropora* gene families. Gene families shared by the two *Montipora* species were defined as *Montipora* gene families. Gene families containing transposon-like genes were excluded from subsequent analyses.

### Transcriptomic comparisons between *Montipora* and *Acropora*

We used RNA-seq data of *M. efflorescens* (planula larvae), *A. tenuis* (blastula, gastrula, planula larvae and polyps) and *A. digitifera* (blastula, gastrula, planula larvae and polyps) (Additional file 7: Table S7). In addition, publicly available RNA-seq data of *M. capitata* (planula larvae, metamorphosed larvae, and recruits) were also used in this study Additional file 7: Table S7). Low-quality reads (quality score < 20 and length < 20 bp) and sequence adaptors were trimmed using CUTADAPT v1.18. Cleaned RNA-seq reads were mapped to each organism's gene models (For *M. capitata* RNA-seq data, we used *M. efflorescens* gene models as a reference) using SALMON v1.0.0 [72]. Expression levels were quantified using SALMON v1.0.0. Genes with TPM > 1 were considered expressed. Then expressed genes were classified into corresponding gene families based on the above gene family inference.

### Estimation of the ratio of nonsynonymous to synonymous substitutions

Protein sequences of putative single-copy orthologs between *M. cactus* and *M. efflorescens* were aligned pairwise with MAFFT [73]. Aligned nucleotide codon sequences without alignment gaps were retrieved using the PAL2NAL script [74]. Genes with nucleotide alignment lengths longer than 120 bp were used for further analysis. We calculated pairwise nonsynonymous (Ka) and synonymous (Ks) substitution ratios of single-copy genes between *M. cactus* and *M. efflorescens* using KaKs_Calculator 2.0 [75] with option “-MA”. Following Villanueva-Canas et al. [76], we discarded gene families showing Ks < 0.01, as such low Ks values may result in inaccurate Ka/Ks estimates, and gene families showing Ks or Ka > 2 indicating saturation of substitutions. Genes exhibiting Ka/Ks ratios with *p* < 0.05 (Fisher’s exact test) were used for further analysis. Subcellular localization of gene families showing Ka/Ks > 1 was predicted using the DeepLoc-1.0 online server [77].

### Statistical analysis

Pairwise proportion tests were conducted to compare lineage-specific gene families (“number of lineage-specific gene families” / “number of gene families in lineage”) and gene annotation proportions of lineage-specific gene families (“number of genes with annotation” / “number of genes without annotation”). Fisher’s exact test was conducted to identify expanded gene families in each group (“number of genes in one gene family in species A” / “number of genes in the rest of the gene family in species A” versus “number of genes in one gene family in species B” / “number of genes in the rest of the gene family in species B”). We considered a *p* < 0.05 as significantly expanded. The Wilcoxon rank sum test was conducted to compare median Ka/Ks values between gene family groups. All statistical tests were performed in R v4.0.3 [78].

## Supplementary Information

**Additional file 1: Table S1.** Summary of comparison of KEGG metabolic pathways among acroporid genomes. MCR indicates the module completion ratio, calculated by GenoMaple.

**Additional file 2: Table S2.** IDs of gene families common to *Montipora* and *Astreopora* or restricted to *Montipora*, that are expressed during three life stages (planula larvae, metamorphosed larvae, and recruits).

**Additional file 3: Table S3.** IDs of gene families common to three genera (*Montipora*, *Acropora* and *Astreopora*) or common to *Montipora* and *Acropora*, that are expressed during three life stages (planula larvae, metamorphosed larvae, and recruits).

**Additional file 4: Table S4.** Summary of gene families significantly (Fisher’s exact test: *p* < 0.05) expanded in the *Montipora* genome compared to those of *Acropora* or *Astreopora*.

**Additional file 5: Table S5.** Summary of samples used for RNA-seq.

**Additional file 6: Table S6.** Summary of publicly available RNA-seq samples that were included for gene prediction in *Montipora* and *Astreopora*.

**Additional file 7: Table S7.** Summary of RNA-seq samples used for comparative transcriptomic analyses.

**Additional file 8: Figure S1.** Summary of gene predictions for *M. cactus*, *M. efflorescens*, and *Astreopora myriophthalma*.

**Additional file 9: Data S1.** Result of orthologous clustering in Acroporidae genomes using OrthoFinder.

**Additional file 10: Data S2.** Summary of retained scaffolds after genome assembly curation for *Montipora cactus *and *M. efflorescens*.

**Additional file 11: Data S3.** Gene models for *M. cactus *in GTF format.

**Additional file 12: Data S4.** Gene models for *M. efflorescens* in GTF format.

**Additional file 13: Data S5.** Gene models for *Astreopora myriophthalma* in GTF format.

## Data Availability

Raw RNA-sequencing data have been deposited in the DDBJ/EMBL/GenBank databases under accession number DRA011820 (BioProject ID: PRJDB11460). A genome browser for *M. cactus*, *M. efflorescens* and *Astreopora myriophthalma* is available from the Marine Genomics Unit web site (https://marinegenomics.oist.jp/gallery). Sequence IDs of retained scaffolds were prepared as Additional file 10: Data S2. Gene models in GTF format for *M. cactus*, *M. efflorescens* and *Astreopora myriophthalma* are provided as Additional file 11: Data S3, Additional file 12: Data S4, Additional file 13: Data S5. For *M. capitata*, we downloaded genome assembly and gene models reported from Shumaker et al. [28] (URL: http://cyanophora.rutgers.edu/montipora/) and Helmkampf et al. [27] (Data set https://doi.org/10.15482/USDA.ADC/1503958).

## Abbreviations

BUSCO: Benchmarking universal single-copy orthologs
KEGG: Kyoto encyclopedia of genes and genomes
DMSP: Dimethylsulfoniopropionate
Ka: Nonsynonymous substitutions
Ks: Synonymous substitutions
TPM: Transcript per million
TRP: Transient receptor potential protein
TTC: Tetratricopeptide repeat protein
CHAT: Caspase HetF associated with Tprs
HOG: Phylogenetic hierarchical orthogroups
MCR: Module completion ratio
