# Supplementary figures and images for: Comparative genomics highlight the importance of lineage-specific gene families in evolutionary divergence of the coral genus, Montipora
Source: BMC Ecol Evol. 2022 May 27;22:71. doi: 10.1186/s12862-022-02023-8 (PMC9145168; doi:10.1186/s12862-022-02023-8)

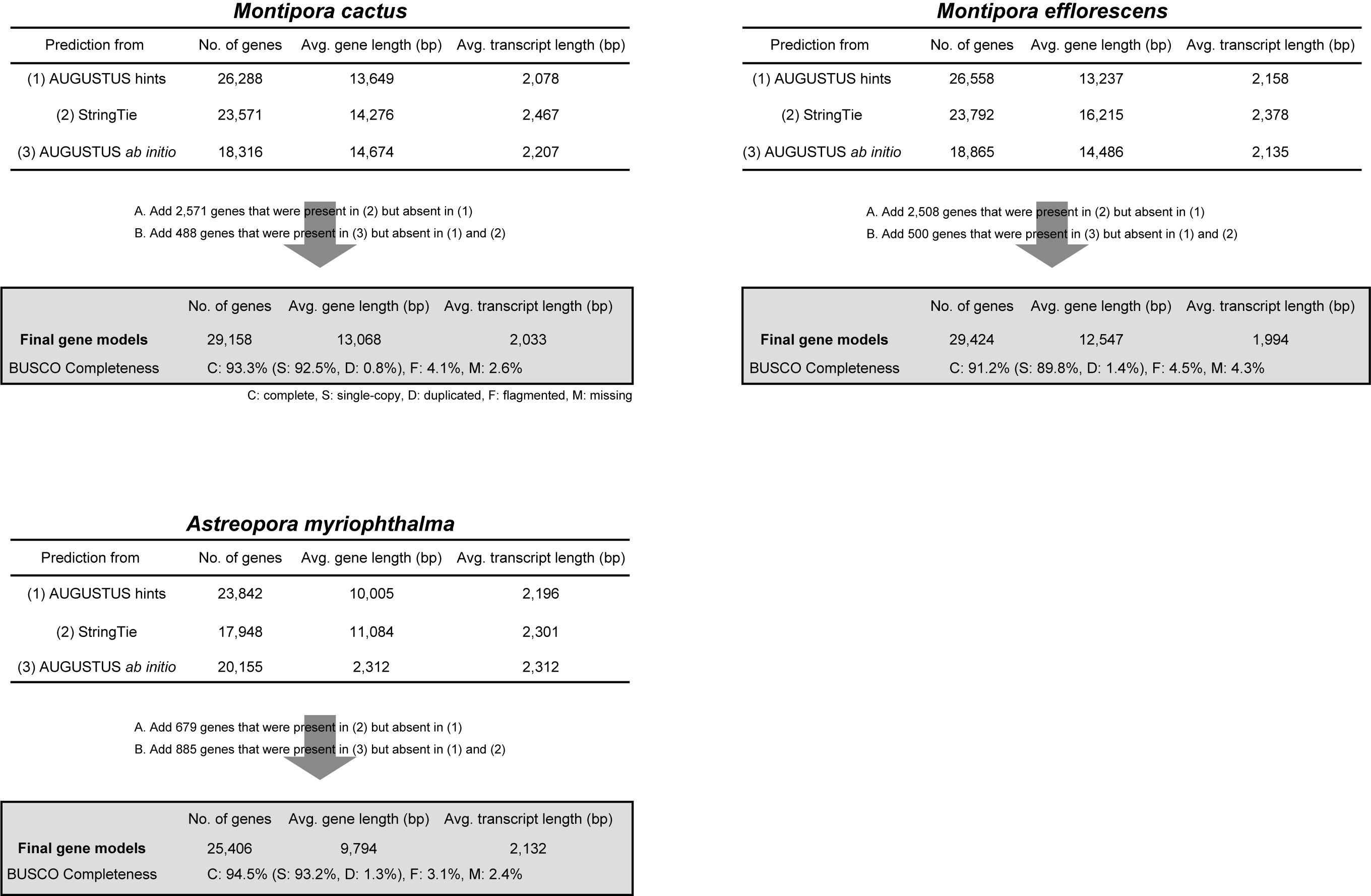

Supplement: Supplementary file 8 — Additional file 8: Figure S1. Summary of gene predictions for M. cactus, M. efflorescens, and Astreopora myriophthalma. [file 12862_2022_2023_MOESM8_ESM.png]
